# Studying gap junctions with PARIS

**DOI:** 10.7554/eLife.45207

**Published:** 2019-03-01

**Authors:** Daniel R Kick, David J Schulz

**Affiliations:** Division of Biological SciencesUniversity of Missouri-ColumbiaColumbiaUnited States

**Keywords:** optogenetics, gap junctions, cardiomyocytes, electrical synapses, olfactory system, *D. melanogaster*

## Abstract

A new genetically encoded system manipulates the pH inside cells to detect whether they are coupled to each other.

**Related research article** Wu L, Dong A, Dong L, Wang SQ, Li Y. 2019. PARIS, an optogenetic method for functionally mapping gap junctions. *eLife* **8**:e43366. doi: 10.7554/eLife.43366

For our bodies to work properly, cells need to communicate with each other. One way to do so is via gap junctions, which are connections between cells that allow small signaling molecules or electrical signals to pass between them. Cells coupled together by gap junctions often synchronize their activity (Bennett and Zukin, 2004).

Neuromodulators and the activity of neurons can affect gap junction coupling between neurons, which, in turn, can influence the pattern of activity in neurons (Lane et al., 2018; Haas et al., 2011). Gap junctions have also been implicated in a variety of diseases (Nielsen et al., 2012). So far, it has been difficult to identify their precise roles in health and disease, as present methods to map or monitor gap junction coupling are often invasive and damaging, or unable to measure a specific cell type in enough detail.

Now, in eLife, Yulong Li and colleagues at Peking University – including Ling Wu as first author – report that they have developed a new method called PARIS to map and measure gap junctions (Wu et al., 2019). PARIS is a genetically encoded system, consisting of an optically controlled ‘actuator’ in one cell (the actuator cell) and a fluorescent sensor in another (the receiver cell). When stimulated with light, the actuator generates an electrochemical gradient with the help of a light-gated proton pump. The activated pump then transports hydrogen ions out of the cell. If the cells are coupled to each other, the departing ions change the pH in the receiver cell, causing a measurable increase in fluorescence (Figure 1A,B).

**Figure 1.**
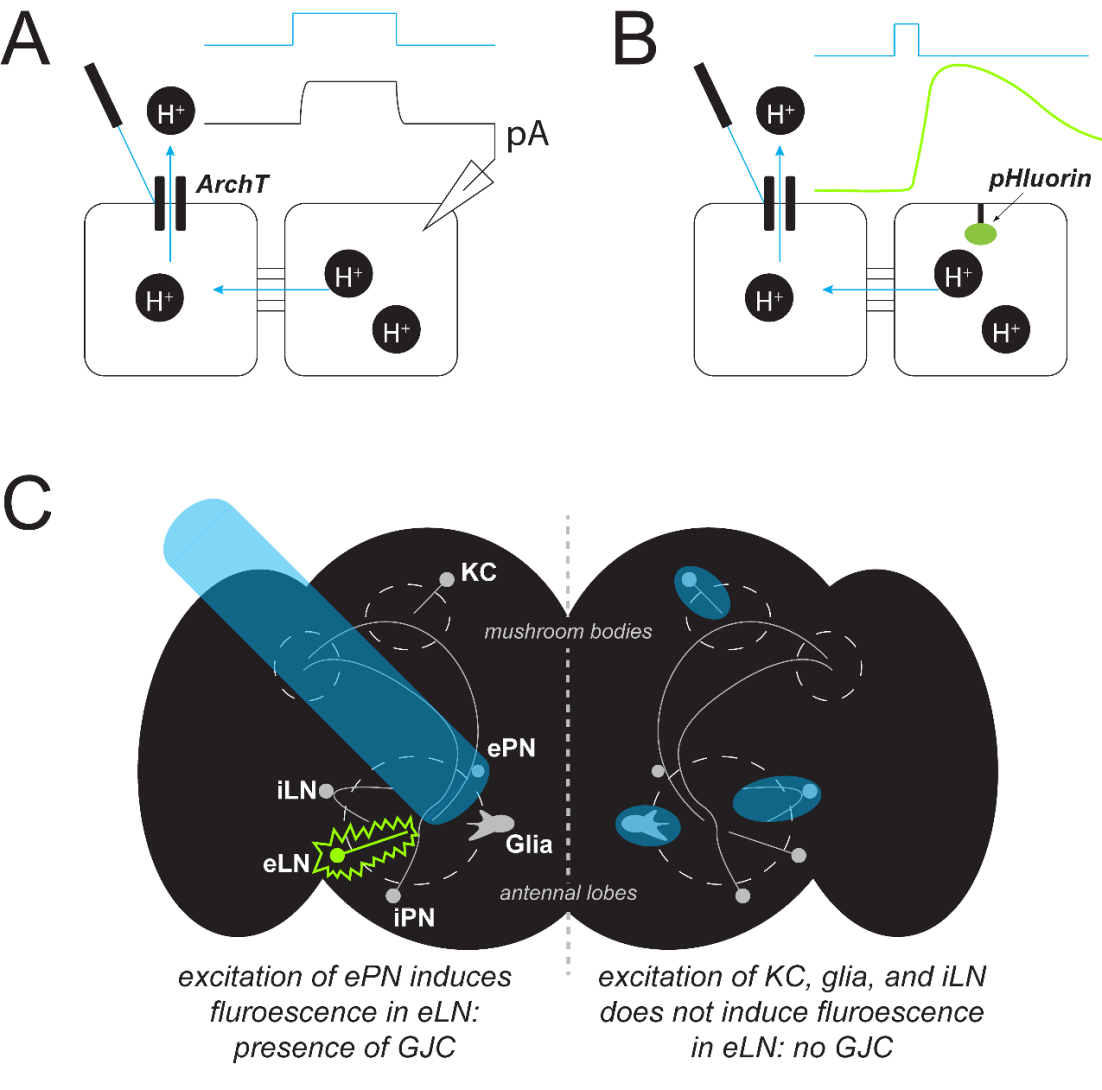
Detecting gap junction coupling. (**A**) A new method called PARIS can be used to study gap junctions between cells (black squares). It is a genetically encoded system in which one cell (the ‘actuator’) contains a light-gated proton pump (ArchT). If the actuator cell is coupled to another (‘receiver’) cell, shining light onto the pump (blue trace) causes protons (H^+^, black circles) to flow, generating a measurable electrical current (black trace, pA). (**B**) In an alternate detection method, a fluorescent sensor (pHluorin, green oval) can be placed in the receiver cell. Once activated, the pump transports protons out of the actuator cell; if the cells are coupled, this induces a change in the pH of the receiver cell that increases the fluorescence of the sensor (green trace). (**C**) PARIS can be used to determine whether gap junction coupling (GJC) exists between different cell types by using light (blue beam) to stimulate a particular type of neuron and checking for fluorescence (green) from another type of neuron. For example, Wu et al. showed that excitatory projection neurons (ePN) in the *Drosophila* brain (black shape, left hemisphere) form gap junctions with excitatory local neurons (eLN) but not with other nearby cell types (KC, glia, iLN).

The pH gradient generated by PARIS can be detected in multiple receiver cells, even when they are not coupled directly to the actuator. Changes to the pH are known to affect the coupling and the strength of gap junctions (Nielsen et al., 2012). However, Wu et al. show that a slight pH increase of 0.1 is sufficient to detect gap junction coupling and therefore does not appear to disrupt the activity of a cell, even in repeated trials.

Furthermore, the change of fluorescence is noticeable within seconds and provides a consistent signal over repeated measurements that is strong enough to be tracked over hours. Moreover, since the technique is fully genetically encoded, there is no need to use dyes or to deliver compounds directly into the cell. Instead, Wu et al. have shown that PARIS can be delivered into the cell by various molecular gene transfer techniques, including virus-based transfer methods, allowing it to be applied to a wide range of organisms.

Given these attributes, PARIS can be used in a variety of fields where it promises to accelerate and expand research. For example, it could be used to study abnormal gap junction coupling associated with certain conditions, or to monitor drugs expected to alter coupling (Haas et al., 2011; Lane et al., 2018; Lane et al., 2016). It may even be used to study signaling within a cell, as it has been shown that certain intracellular enzymes can alter gap junction coupling too.

The ability of PARIS to assess gap junction coupling between individual cell types, especially in the brain, is particularly attractive. Wu et al. were able to confirm that in the olfactory system of fruit flies, a type of neuron known as an excitatory projection neuron, forms gap junctions with excitatory local neurons, but not with other nearby cell types (Figure 1C; Huang et al., 2010; Yaksi and Wilson, 2010).

PARIS also can be used to gain insight into where cells connect with each other. For example, the researchers revealed that excitatory and inhibitory projection neurons are coupled at their dendrites rather than at their axons. This degree of resolution is invaluable for studying nervous systems, and is difficult to achieve by other means.

Projects to map the brain connectivity in humans and other species based on chemical synaptic connectivity alone will be incomplete because they fail to describe alternate pathways information may use to travel, such as gap junctions (Marder et al., 2017). With future improvements in the sensitivity of the fluorescent receiver to an even smaller pH change, PARIS has the potential to be a valuable tool to study cell communication and its role in many different diseases.
